# Botryomycosis: series of cases diagnosed between 2000 and 2023 in the Dermatology division of a tertiary hospital^؆^

**DOI:** 10.1016/j.abd.2025.501144

**Published:** 2025-07-07

**Authors:** Nicole de Souza Aranha, João Avancini, Caroline Heleno Chagas de Carvalho, Walter Belda, Marcello Menta Simonsen Nico

**Affiliations:** Department of Dermatology, Faculty of Medicine, Universidade de São Paulo, São Paulo, Brazil

*Dear Editor,*

Botryomycosis is a rare chronic granulomatous disease that can present as a cutaneous or visceral disease.^1^ It is important to differentiate it from other diseases that produce grains as a parasitic form of the etiological agent, including eumycetoma and actinomycosis, aiming targeted and assertive treatment, preventing disease progression and ensuring better quality of life for the affected individuals.

Therefore, the objective of this case series is to present seven cases of botryomycosis treated at a university dermatology service, from January 2000 to 2023, analyzing the patient’s individual characteristics, locations of the lesions, therapeutic approaches, and responses to the proposed treatments.

Table 1 summarizes the main information of the cases. Seven patients were followed, five males and two females. The ages at onset of the condition ranged from 17 to 79 years. Five of the cases had lesions on the feet, one on the leg, and one on the dorsum of the hands and fingers. Only three cases reported previous trauma. One patient had a history of Cushing’s syndrome secondary to a pituitary microadenoma, which was excised through transsphenoidal surgery. Another patient had a history of immunosuppression with 38 radiotherapy sessions due to prostate cancer; however, after the onset of the skin condition. Two cases had other comorbidities such as systemic arterial hypertension, congestive heart failure, hypothyroidism, and dyslipidemia. Three cases had no comorbidities.

**Table 1:** Summary of botryomycosis cases.

Age	Sex	Location	Agent	Treatment	Evolution
17	Male	Hands	*Staphylococcus aureus*	Sulfamethoxazole and Trimethoprim	Improvement
33	Male	Foot	*Staphylococcus aureus*	Sulfamethoxazole and Trimethoprim	Improvement
17	Male	Foot	*Staphylococcus aureus*	Multiple antibiotic regimens	Progressive worsening, requiring amputation
17	Male	Foot	*Staphylococcus aureus*	Sulfamethoxazole and Trimethoprim	Improvement
79	Male	Pre-tibial	*Staphylococcus aureus*	Sulfamethoxazole and Trimethoprim	Loss to follow-up
44	Female	Foot	*Staphylococcus warneri*	Sulfamethoxazole and Trimethoprim	Improvement and recurrence
34	Female	Foot	Not reported	Surgical resection and Sulfamethoxazole and Trimethoprim	Loss of follow up

The majority presented clinically with nodules and tumors with fistulous tracts and three cases had grain discharge. None of the patients had systemic symptoms, except for one who developed anemia and thrombocytopenia due to continued bleeding from the lesion.

The isolated microorganism was *Staphylococcus aureus* in six cases, whereas *Staphylococcus warneri* was repeatedly cultured in one case.

In all cases, treatment was carried out with the use of sulfamethoxazole associated with trimethoprim (800 + 160 mg) for approximately six months to two years. Three patients responded well and two lost follow-up. In one case, surgical resection was proposed in combination with amikacin, but the patient did not agree to the procedure and did not return for follow-up appointments. One patient did not respond to all antibiotic and antifungal treatments, and after two years, transtibial amputation was necessary due to mobility difficulties and blood loss from the lesion.

Botryomycosis is a rare chronic granulomatous disease caused by non-filamentous bacteria, the main one being *Staphylococcus aureus*. Other bacteria occasionally involved are *Pseudomonas* spp., *Escherichia coli*, *Proteus* spp. and *Streptococcus* spp. The term botryomycosis derives from the Greek “botrys” = grains; “mycosis” = fungus, as it was believed that the condition was of fungal origin.^1^, ^2^

The disease pathogenesis is not well established; it is believed that an antigen-antibody complex, immunoglobulin G and C3 are precipitated around the microorganisms, preventing phagocytosis and intracellular bacterial destruction.^1^

The condition generally affects areas prone to trauma and may present as cutaneous or visceral disease.^3^ The cutaneous form manifests clinically as tumors or plaques that show fistulous tract openings on their surface that drain white grains, which may invade deep tissues, leading to extensive local destruction.^4^, ^5^, ^6^

Diagnosis includes clinical suspicion, histopathological and microbiological studies. It is important to differentiate it from other diseases that produce grains as a parasitic form of the etiological agent, including eumycetoma and actinomycosis (Fig. 1, Fig. 2, Fig. 3, Fig. 4).^2^, ^5^

**Fig. 1:**
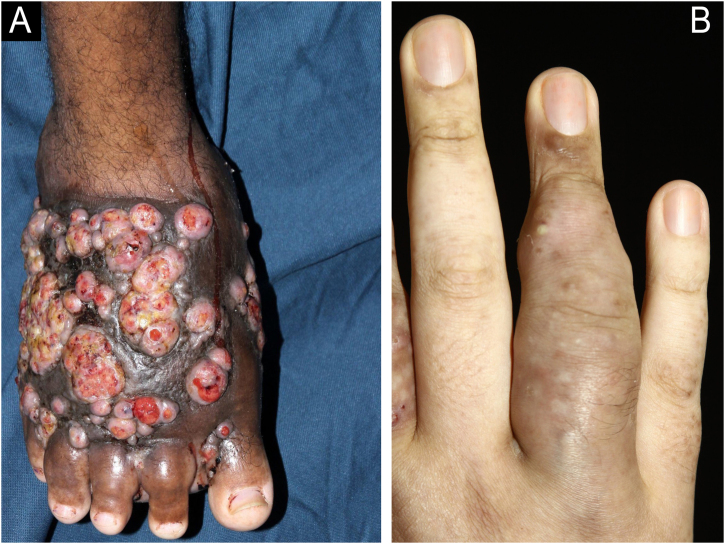
Clinical. (A) Tumor showing fistulae in different stages of evolution located on the dorsum of the right foot. (B) Nodules and fistulae on the fingers.

**Fig. 2:**
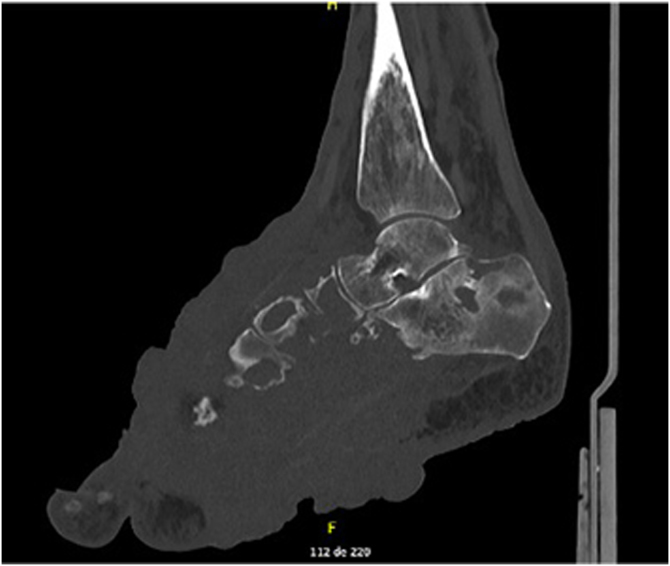
Computed tomography of the right foot showing voluminous expansive and infiltrative solid tissue with extensive bone and soft tissue involvement.

**Fig. 3:**
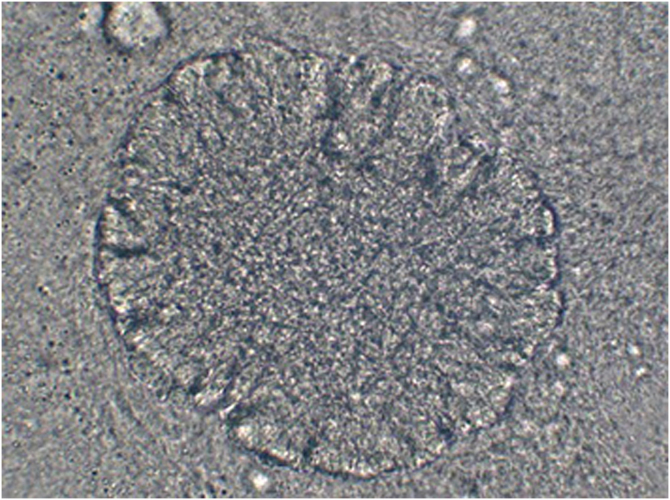
Botryomycosis grain analyzed through direct microscopic examination, showing aggregates of cocci.

**Fig. 4:**
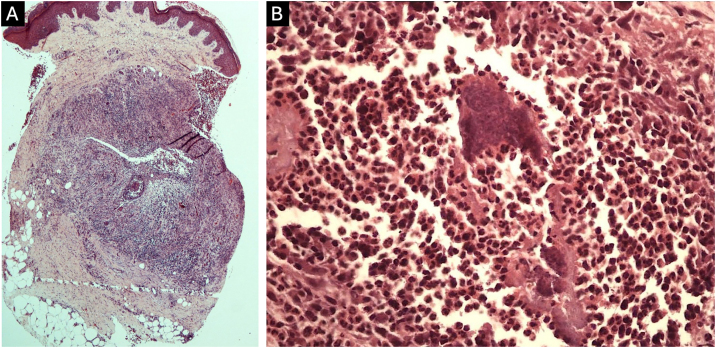
Histopathology. (A) Histological section showing chronic suppurative granulomatous dermatitis containing grain in the deep dermis (Hematoxylin & eosin, ×40). (B) Rounded structures forming a grain, are involved by the Splendore-Hoeppli phenomenon (Hematoxylin & eosin, ×400).

The histopathological examination allows the identification of the microorganism type, since the grains consist of true fungi in eumycetoma, whereas in actinomycetoma it is possible to visualize filamentous bacteria and the botryomycosis grains consist of non-filamentous bacteria. Histochemical stains such as Grocott and Gram are useful for the characterization of fungi and bacteria.^2^

Treatment involves targeted antibiotic therapy, surgical approach with local debridement, and, in selected cases, when there is antibiotic response failure and when the limb becomes dysfunctional, amputation of the affected limb may be necessary, as occurred in one of the presented cases.^2^

Most publications on the subject are case reports and, due to its rarity, there are few published series. Early diagnosis of botryomycosis should favor prognosis and response to treatment since extensive cases with large volume and fibrosis show poor response to therapeutic agents.

## Authors' contributions

Nicole de Souza Aranha: Design and planning of the study; collection of data; drafting and editing of the manuscript; interpretation of data; critical review of the literature; approval of the final version of the manuscript.

João Avancini: Analysis and interpretation of data; effective participation in research orientation; intellectual participation in the propaedeutic and/or therapeutic conduct of the studied cases; approval of the final version of the manuscript.

Caroline Heleno Chagas de Carvalho: Analysis and interpretation of data; effective participation in research orientation; intellectual participation in the propaedeutic and/or therapeutic conduct of the studied cases; approval of the final version of the manuscript.

Walter Belda Júnior: Analysis and interpretation of data; effective participation in research orientation; intellectual participation in the propaedeutic and/or therapeutic conduct of the studied cases; approval of the final version of the manuscript.

Marcello Menta Simonen Nico: Design and planning of the study; analysis and interpretation of data; critical review of important intellectual content; effective participation in research orientation; intellectual participation in the propaedeutic and/or therapeutic conduct of the studied cases; approval of the final version of the manuscript.

## Financial support

None declared.

## Conflicts of interest

None declared.
